# Cystoscopy-assisted laparoscopy for bladder endometriosis: modified light-to-light technique for bladder preservation

**DOI:** 10.1590/S1677-5538.IBJU.2014.0362

**Published:** 2017

**Authors:** Rafael Mamprin Stopiglia, Ubirajara Ferreira, Daniel Gustavo Faundes, Carlos Alberto Petta

**Affiliations:** 1 Grupo de Urologia Oncológica, Universidade de Campinas, UNICAMP, SP, Brasil;; 2 Centro de Reprodução Humana Campinas, SP, Brasil;; 3 Departamento de Ginecologia, Universidade de Campinas, UNICAMP, SP, Brasil

**Keywords:** Endometriosis, Urinary Bladder, Cystoscopy

## Abstract

**Introduction:**

Endometriosis is a disease with causes still unclear, affecting approximately 15% of women of reproductive age, and in 1%-2% of whom it may involve the urinary tract. The bladder is the organ most frequently affected by endometriosis, observed around 85% of the cases. In such cases, the most effective treatment is partial cystectomy, especially via videolaparoscopy.

**Study Objective, Design, Size and Duration:**

In order to identify and delimit the extent of the intravesical endometriosis lesion, to determine the resection limits, as well as to perform an optimal reconstruction of the organ aiming for its maximum preservation, we performed a cystoscopy simultaneously with the surgery, employing a modified light-to-light technique in 25 consecutive patients, from September 2006 to May 2012.

**Setting:**

Study performed at Campinas Medical Center – Campinas – Sao Paulo – Brazil.Participants/materials, setting and methods: Patients aged 27 to 47 (average age: 33.4 years) with deep endometriosis with total bladder involvement were selected for the study. The technique used was conventional laparoscopy with a transvaginal uterine manipulator and simultaneous cystoscopy (the light-to-light technique). A partial videolaparoscopic cystectomy was performed with cystoscopy-assisted vesical reconstruction throughout the entire surgical time. The lesions had an average size of 2.75cm (ranging from 1.5 to 5.5cm). The average surgical time was 137.7 minutes, ranging from 110 to 180 minutes.

**Main Results:**

Postoperative follow-up time was 32.4 months (12-78 months), with clinical evaluation and a control cystoscopy performed every six months. No relapse was observed during the follow-up period.

**Conclusions:**

A cystoscopy-assisted partial laparoscopic cystectomy with a modified light-to-light technique is a method that provides adequate identification of the lesion limits, intra or extravesically. It also allows a safe reconstruction of the organ aiming for its maximum preservation.

## INTRODUCTION

Endometriosis is a gynecologic disease with causes still unclear. It was first described in 1860, but its most accepted etiopathogeny postulating retrograde menstruation was proposed in 1921 (1, 2).

Endometriosis is the presence of stroma and/or endometrial epithelium outside the cavity and the uterine muscles, invading the peritoneum or embedding on the walls of the pelvic organs (3). It is an estrogen-dependent disorder associated with chronic pelvic pain and infertility (4).

It is estimated that approximately 15% of women of reproductive age are affected by endometriosis (5).

In 1979, the American Fertility Society initially classified endometriosis in 4 stages of severity, but reviewed this classification in 1985 (6, 7). The present classification was introduced in 1997, whereby it stages endometriosis as superficial when it affects the parietal and visceral layers of the peritoneal membrane, and deep when there is more than 5cm penetration of the walls of the organs (8).

The most common sites affected by endometriosis in the pelvic cavity are the torus uterinus, the posterior fornix, the uterosacral ligaments, the rectum, the vagina and the urinary tract (9). However, it may affect other sites, such as the diaphragm, the umbilical cord, the ileum, the lungs, the pleura, the pericardium and the brain (10, 11).

Endometriosis may cause dysmenorrhea, even at the beginning of a woman´s fertile age, dyspareunia, chronic pelvic pain, and peri-menstrual pain (12).

Another frequent disorder is infertility, occurring in up to 60% of the cases.

Specifically, in the urinary tract, there is a 0.3% to 12% incidence of endometriosis; however, it is usually reported as 1%-2% (13), and the most commonly affected sites are the bladder (85%), ureter (9%), kidneys (4%), and the urethra (2%), as shown in Figure 1A below.

**Figure 1A:**
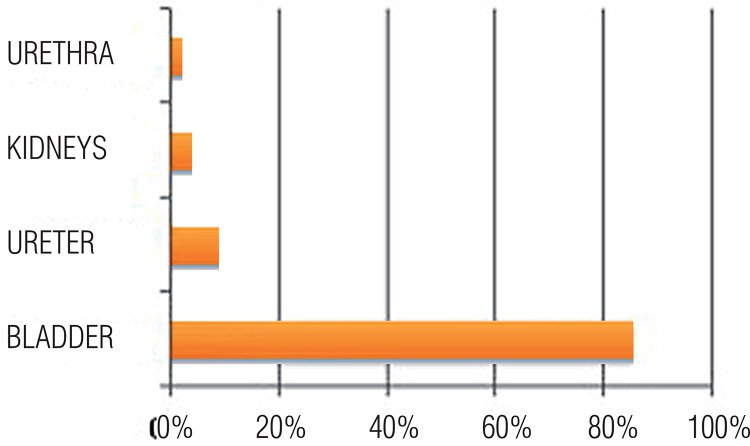
Endometriosis incidence in utrinary tract

When the bladder is affected, 70% of women present pain during urination, dysuria, suprapubic pain and hematuria, especially during the peri-menstrual period.

There is a 20%-35% occurrence of hematuria due to vesical mucous infiltration. Menouria (hematuria during the menstrual period) is infrequent (14).

Urinary tract involvement may be represented by nodules with retractions and/or distortions of the normal anatomy (15), in addition to adherences to the vesico-uterine space.

Partial cystectomy – especially by laparoscopic means - is the most effective treatment for deep endometriosis when the bladder is affected. This surgical procedure is excisional and consists of the removal of the entire bladder wall affected by endometriosis. For this type of procedure, the bladder must ideally present good functional capacity, show a single lesion and be located >5mm of the urethral meatus.

## PATIENTS AND METHODS

From September 2006 to May 2012, 25 patients with initial diagnosis of deep endometriosis affecting the bladder wall were treated by the cystoscopy-assisted videolaparoscopic cystectomy with the light-to-light technique (16). (The association of both procedures is meant to identify and delimit the extent of the intravesical endometriotic lesion, to determine the resection limits, as well as to perform an optimal reconstruction of the organ, aiming for its maximum preservation. The patient’s average age was 33.4 years, ranging from 27 to 47 years. After clinical assessment and a physical examination with bimanual palpation, the patients were tested for serum urea and creatinine levels, urine (proteinuria or microscopic hematuria) and urine culture, all of which were normal. All patients were submitted to transvaginal ultrasound (TVUS) to diagnose the disease (Figure-1B), and to magnetic resonance (MRI) of the pelvis for surgical planning purposes (Figure-1C). The vesical lesion depicted on MRI is characterized by hyper-signal on T1 and hypo-signal on T2 (17, 18).

**Figure 1B:**
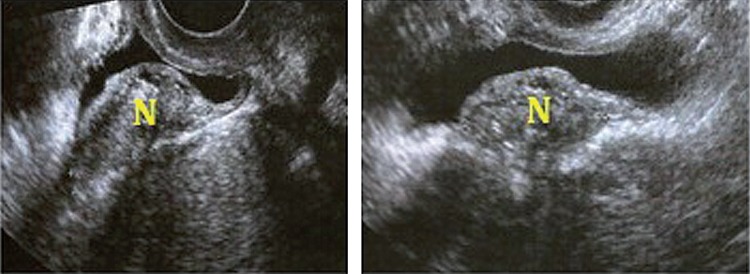
Transvaginal ultrasound with endometriotic endovesical lesion. (N)

**Figure 1C:**
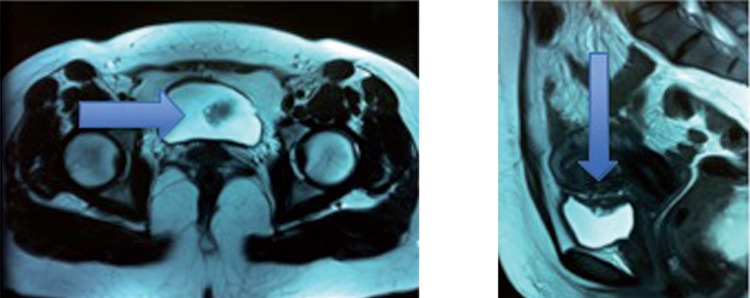
MRI of the pelvis depicting hypo-signal on T2 (lesion is highlighted).

### Description of the Modified Light-To-Light Technique

The technique we used consisted of conventional laparoscopy, with the patient under general anesthesia and in a horizontal supine (dorsal decubitus) position, with the lower limbs spread out for the cystoscopy procedure. The umbilical scar is punctured with a Veress needle and pneumoperitoneum is performed with CO2 initially up to 20mmHg until introduction of a 10mm umbilical trocar. Upon visibility of the abdominal cavity, the pressure is reduced to up to 12mmHg and 3 trocars are introduced, of which one 10mm trocar in the umbilical scar, one 10mm trocar in the bisector of the imaginary line going from the anterior superior iliac crest to the umbilical scar on the right, and one 5mm in the exact same position on the left side, as per the representation below.

A videolaparoscopy subsequently performed inventory of the abdominal and pelvic cavity and identified a solid nodular lesion on the vesical dome and vesico-uterine fossa, at times with significant adherence of such organs, as shown in Figure-1D(a) .

**Figure 1D:**
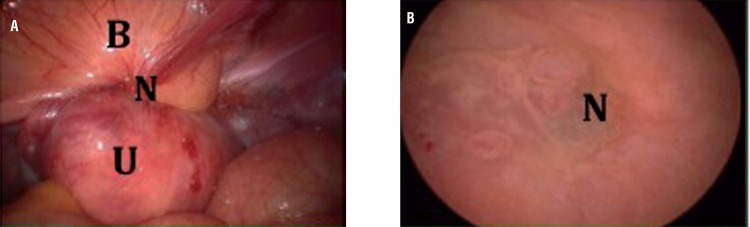
A - Endometriosis in laparoscopic view, B - Endometriosis in cistoscopic view.

Figure 1D, **(a)** (left): laparoscopic view; (**B**=Bladder, **N**=Node, **U**=Uterus); Figure 1D, **(b)** (right): cystoscopic view; (N=node)

The procedure above was followed by positioning the transvaginal uterine manipulator and performance of the light-to-light cystoscopy technique, originally described by Seracchioli et al. The endoscopic diagnosis was confirmed by visualization of tissue compatible with endometriosis on the vesical mucous surface. These lesions were blistered purple-blue nodules, containing endovesical material, as shown in Figure1D(b).

The cystoscopy-assisted partial laparoscopic cystectomy with the light-to-light technique was then performed with some modifications, such as initially not inserting urethral catheters. As the lesions affected the entire bladder wall, a partial cystectomy was performed assisted by cystoscopic visualization throughout the procedure. Both surgeons identified and delimited the lesion, keeping a margin of at least 5mm of healthy tissue. Biopsies of the lower, right lateral, left lateral and superior margins were performed after exeresis of the lesion to eliminate permanence of the disease. The subsequent vesical reconstruction consisted of a one-layer suture with monofilament absorbable 3.0 thread, with continuous cystoscopy monitoring, to ensure better visualization of the suture and final checking of the procedure, thus allowing maximum possible preservation of the healthy vesical tissue (Figure 1E).

**Figure 1E:**
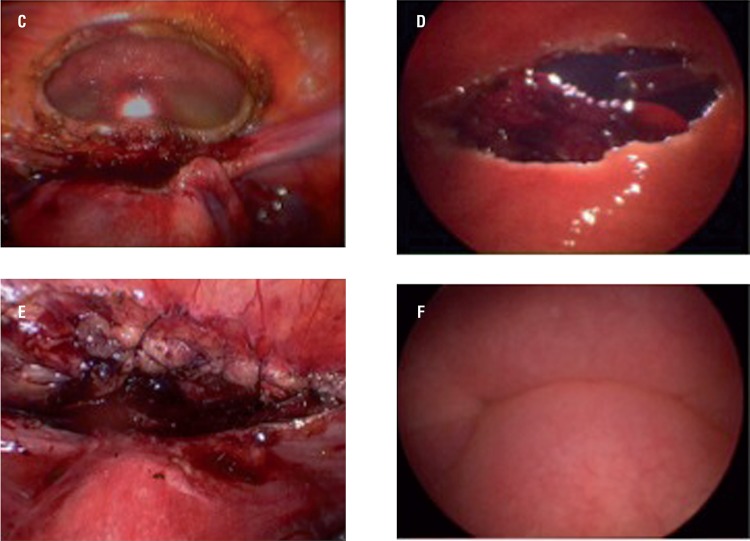
C e E - Endometriosis ressection and suture in laparoscopic view D e F - Endometriosis ressection and suture in cistoscopic view.

Clockwise: **c =** partial cystectomy, laparoscopic view; **d =** partial cystectomy, cystoscopic view; **e =** cystorraphy, laparoscopic view; **f =** cystorraphy, cystoscopic view.

All patients maintained a urethral catheter for 7 days.

## RESULTS

Of the 25 treated patients, 15 had already undergone previous laparoscopy for treatment of pelvic endometriosis and endometriomas, and 10 had never had any treatment. Surgical time ranged from 110 to 180 minutes, with an average of 137.7 minutes. The resected lesions varied in size, ranging from 1.5 to 5.5cm, with an average of 2.75cm. No significant bleeding was observed and average length of hospital stay was 24 hours.

Follow-up was made every six months by means of clinical assessment and a cystoscopy, with total follow-up time of 32.4 months in average (ranging from 12 to 78 months) (Table-1).

**Table 1:** Date table.

Patients	Age (Years)	Nodule (CM)	Surgical Time (Minutes)	Follow-UP (Months)
1	30	2.0	180	78
2	27	3.0	180	72
3	28	2.5	172	60
4	33	1.8	175	50
5	29	2.2	168	45
6	42	3.3	150	42
7	42	3.7	155	41
8	36	4.0	160	36
9	36	2.5	120	34
10	29	2.7	120	33
11	33	3.0	132	29
12	31	3.3	128	29
13	34	2.5	124	28
14	47	2.0	120	26
15	33	1.5	110	26
16	30	2.0	122	25
17	32	2.7	128	24
18	33	5.5	150	24
19	29	2.8	127	20
20	28	1.7	118	18
21	30	2.5	120	18
22	35	3.1	115	15
23	38	3.5	126	14
24	39	2.2	111	12
25	32	2.8	122	12

AVERAGE	33.4 years	2.75 cm	137.7 minutes	32.4 months

There was no relapse of the disease in all cases. The patients presented normal vesical physiology without alterations in bladder filling or emptying, evidenced by clinical assessment and cystoscopy.

## DISCUSSION

The morpho-physiology of the vesical endometriosis lesions may vary according to the menstrual cycle. However, the lesions are better identified during menstruation. At cystoscopy, these lesions may appear in several colors, such as shades of red, blue, brown or even black. The urothelium is usually rarely ulcerated (19).

Biopsies for differential diagnosis with urothelial carcinoma have been described, but since it rarely invades the mucous, it is difficult to reach a diagnosis by this means.

The differential diagnoses are hyperactive bladder, interstitial cystitis (painful bladder syndrome), urethral syndrome and urothelial carcinoma (20).

In patients with clinical suspicion, diagnosis may be made via a transvaginal ultrasound and, in some cases, by magnetic resonance, as previously described. However, the most effective diagnostic method, whether for superficial or deep lesions, is laparoscopy (21).

Treatment of pelvic endometriosis affecting the bladder may depend on several factors, such as age, symptom intensity, fertility, extent of the disease, presence in other organs and level of menstrual dysfunction. As the disease originates outside the bladder (in the peritoneum), subsequently invading it, a vesical transurethral resection is usually an ineffective method (22, 23).

The disease is hormone (estrogen)-dependent, therefore the treatment of superficial lesions is based on hormonal blockade. The most commonly adopted therapy for this purpose is the association of GnRH analogues, progestogens and oral contraceptives (24). This treatment aims at temporarily suppressing endometriosis, reason why it is more recommended for younger patients without deep endometriosis who wish to preserve their fertility. An intrauterine device (IUD) with levonorgestrel may also be used in these more conservative cases, in addition to acting as an adjuvant in corrective surgeries. The IUD importance rests on the fact that is has a duration of up to 5 years and maintains fertility upon discontinuance of its use (25).

There are some options available for cases of deep vesical endometriosis, depending on the extent and site of the lesion in relation to its distance from the urethral meatus.

A transurethral resection with simultaneous use of analogues may be performed. However, the relapse rates in such cases are of approximately 25%-35%, and there are high rates of vesical perforation in diseases of greater extension (26).

Therefore, better results are obtained with partial cystectomy in terms of cure of the disease, whether the approach is open, laparoscopic or robotic-assisted (27, 28), with conventional laparoscopic partial cystectomy being the method of choice (27). Several studies report surgical results with 95%-100% symptom remission rates and low rates of relapse (28).

The simultaneous association of cystoscopy with laparoscopy may guide the surgeon in terms of laparoscopic identification of the lesion, with better visibility of the vesico-uterine space, identification and dissection of the nodule, allowing exeresis of its total extension, and verification of the margins free of the disease.

Healthy 5mm margins of the bladder and a distance of at least 1cm of the urethral meatus should ideally be preserved (29).

The laparoscopic approach has as advantages less blood loss, less time of hospital stay, less use of pain killers and better aesthetic results (30).

We also agree that the interaction between gynecologists and urologists is relevant for the best treatment of this disease and for the performance of successful procedures.

## CONCLUSIONS

A cystoscopy-assisted partial laparoscopic cystectomy with a modified light-to-light technique is a method that provides adequate identification of the lesion limits, intra or extravesically. It also allows a safe reconstruction of the organ aiming at its maximum preservation.

## ARTICLE INFO

Int Braz J Urol. 2017; 43: 87-94
